# Sterculic Oil, a Natural SCD1 Inhibitor, Improves Glucose Tolerance in Obese ob/ob Mice

**DOI:** 10.5402/2012/947323

**Published:** 2012-11-14

**Authors:** Laura C. Ortinau, R. Taylor Pickering, Karen J. Nickelson, Kelly L. Stromsdorfer, Chaitasi Y. Naik, Rebecca A. Haynes, Dale E. Bauman, R. Scott Rector, Kevin L. Fritsche, James W. Perfield

**Affiliations:** ^1^Department of Nutrition and Exercise Physiology, University of Missouri, Columbia, MO 65211, USA; ^2^Department of Food Science, University of Missouri, Columbia, MO 65211, USA; ^3^Department of Animal Science, Cornell University, Ithaca, NY 14853, USA; ^4^Department of Internal Medicine-Division of Gastroenterology and Hepatology, University of Missouri, Columbia, MO 65211, USA; ^5^Division of Research Service, Harry S. Truman Memorial Veterans Medical Center, Columbia, MO 65201, USA; ^6^Department of Animal Sciences, University of Missouri, Columbia, MO 65211, USA

## Abstract

Obesity and its metabolic complications are associated with increased expression/activity of stearoyl-CoA desaturase-1 (SCD1), a major regulator of lipid metabolism. Reduction or ablation of this enzyme is associated with an improved metabolic profile and has gained attention as a target for pharmaceutical development. Sterculic oil (SO) is a known inhibitor of SCD1 and may provide a natural approach for treating obesity and/or insulin resistance. The purpose of this study was to evaluate the effects of SO consumption in leptin-deficient ob/ob mice, a model of obesity and insulin resistance. Five-week-old male mice received either an AIN-93G (control) or an AIN-93G diet containing 0.5% SO. After 9 weeks, SO supplementation did not alter food intake or body weight; however, the desaturase indices, a proxy of SCD1 activity, were reduced in liver and adipose tissue of SO-supplemented animals. This reduction was associated with improved glucose and insulin tolerance and attenuated hepatic inflammation in obese ob/ob mice, while no appreciable changes were observed in lean control mice receiving SO. Future studies are needed to better understand the mechanism(s) by which SO is functioning to improve glucose metabolism and to further explore the nutraceutical potential and health implications of SO supplementation.

## 1. Introduction

Obesity is an epidemic in the United States, with approximately one third of adults and 17% of children and adolescents being obese [1].Obesity is often associated with the onset of insulin resistance and the eventual development of type 2 diabetes [2, 3]. In an attempt to treat obesity and improve insulin sensitivity, numerous dietary, pharmaceutical, and lifestyle interventions have been investigated [4, 5]. Currently, there is increased interest in dietary supplements containing nutraceuticals that have been determined to be beneficial to the human body either by preventing chronic diseases or improving metabolic health [4, 5]. The most recent NHANES data indicate that just over half of the U.S. population regularly consumes dietary supplements [6].

Approximately 55% of diabetic patients use dietary supplements including cinnamon, resveratrol, and multivitamins as a complementary therapy to treat their diabetes [7, 8]. A product that has yet to be thoroughly examined for its potential nutraceutical properties is sterculic oil (SO), a known natural inhibitor of stearoyl-CoA desaturase 1 (SCD1) [9, 10]. Sterculic oil contains two unique cyclopropenoic fatty acids, sterculic (55%), and malvalic (10%) acids [11, 12], which inhibit the enzymatic activity of SCD1 [9, 10]. Recently, SCD1, a major enzyme involved in fatty acid metabolism, has emerged as a potential pharmaceutical target for the treatment of obesity and type 2 diabetes [13, 14]. Elevation of SCD1 activity is associated with increased obesity and the metabolic syndrome in humans [15, 16], while transgenic mice lacking SCD1 are protected from developing obesity induced insulin resistance [17, 18]. Therefore, inhibition of SCD1 by a natural product may also provide health benefits. 

However, in order to protect the consumer, a need exists to rigorously evaluate potential nutraceutical products. The health claims associated with these products should be based on scientific data demonstrating a biological effect (improved health or reduced disease risk) as well as a mechanism(s) of action. *In vivo* and *in vitro* studies have confirmed the ability of sterculic oil to attenuate SCD1 activity [9, 10, 19, 20]. Nonetheless, the metabolic consequences of SO consumption remain unclear. A limited number of studies with a range of experimental paradigms have reported both beneficial and potentially adverse outcomes when SO was consumed [9, 21–25]. Therefore, the purpose of the present study was to better understand the metabolic changes associated with SO consumption in lean and obese mice by evaluating its ability to inhibit SCD1 activity and prevent the onset of obesity and/or insulin resistance in ob/ob mice.

## 2. Materials

### 2.1. Animals and Animal Care

The University of Missouri Animal Care and Use committee approved all procedures involving mice. Animals were maintained at a controlled temperature (22°C) and a 12 hr light : 12 hr dark cycle. Five week old male ob/ob and C57BL/6 mice were individually housed and assigned to one of two dietary treatment groups for 9 wks: (1) an AIN-93G diet (WT AIN; OB AIN) or (2) an AIN-93G diet supplemented with 0.5% SO (WT SO; OB SO). Diet composition and fatty acid profiles for diets are presented in Supplemental Tables 1 and 2, respectively; available online at doi:10.5402/2012/947323. Food intake and body weight were measured weekly.

### 2.2. Measurements of Glucose Homeostasis

At 7 wks on diet, an insulin tolerance test (ITT) was performed after a 6 hr fast. Initially, a baseline blood sample was taken from the tail vein at time point zero. Then an i.p. injection of insulin (1.0 U/kg BW) was administered and blood glucose concentrations were determined using a handheld glucometer at 15, 30, 45, 60, and 90 min post injection. At 8 wk on diet, animals were fasted for 6 hr and a glucose tolerance test (GTT) was performed. A baseline blood glucose sample was taken from the tail, then sterile glucose (1 g/kg body weight) was injected i.p., and blood glucose concentrations were determined at 30, 60, 90, and 120 min post injection. 

### 2.3. Tissue Collection and Histological Analysis

At 9 wk on diet, animals were fasted 10–12 hr, and blood glucose was measured. Animals were then euthanized by CO_2_ asphyxiation followed by exsanguination via cardiac puncture. Plasma was separated by centrifugation, aliquoted, and frozen for future analysis. The liver and individual adipose depots (gonadal, subcutaneous, and perirenal) were excised, weighed, and snap frozen for analysis. A portion of the adipose tissues were fixed in 4% paraformaldehyde, embedded in paraffin, and sectioned for histological analysis. Sections of the gonadal and subcutaneous adipose depots were then stained with hematoxylin and eosin (H and E staining), and digital images were acquired using an Olympus BX51 light microscope and Olympus DP70 camera. Adipocyte volume was calculated using the cross-sectional area obtained from perimeter tracings using Image J software (Sun Microsystems, Santa Clara, CA).

### 2.4. Real-Time Quantitative PCR

Total mRNA was extracted from liver and adipose tissue using RNeasy mini and RNeasy lipid tissue kits with on-column DNase digestion (Qiagen), respectively. Purity and concentration were determined with a Nanodrop 1000 spectrophotometer (Thermo Scientific). 1 *μ*g of RNA was used to synthesize cDNA with a reverse transcriptase polymerase chain reaction kit (Applied Biosystems) and diluted to 10 ng/*μ*L. Expression of mRNA was determined using SYBR green qRT-PCR on an Applied Biosystems StepOne Plus RT-PCR system. Fold difference for gene expression was calculated as 2^−ΔΔCT^ using the endogenous control genes Cyclophilin b (liver), RPS-3 (gonadal adipose tissue), or 18S (subcutaneous adipose tissue).

### 2.5. Tissue Fatty Acid and Triglyceride Analysis

Tissue fatty acids were extracted using a modified version of Folch and Bligh and Dyer. Briefly, tissues were homogenized in a 50 mM Trizma hydrochloride and 1 mM EDTA-disodium salt solution and chloroform/methanol/acetic acid (2 : 1 : 0.015, v/v/v) was added to create a phase separation. The top organic layer was isolated, dried under N_2_, and methylated using 0.5 N sodium methoxide [26]. Fatty acid methyl esters were analyzed using a gas chromatograph (Varian Star 3400) equipped with a 100 m, 0.25 mm I.D., and 0.20 um film column (Supelco, Bellefonte, PA). Gas chromatograph conditions were a helium flow rate of 1 mL/min with an initial temperature of 140°C held for 5 min. The column temperature was then increased to 250°C at a rate of 2°C/min and held at 250°C for 15 min. Fatty acid peaks were identified using pure methyl ester standards (Nu-Chek Prep, Elysian, MN).

 Liver triglyceride content was determined using a modified protocol described by Schwartz and Wolins [27]. Briefly, powdered liver (~30 mg) lipid was extracted using a chloroform/methanol (1 : 2, v/v) and 4 mM MgCl solutions. The organic phase was then separated, dried, reconstituted in butanol-Triton X-114 (3 : 2, v/v), and vortexed. Triglyceride was then quantified using a colorimetric enzyme-linked kit (Sigma; St. Louis, MO) and concentration was expressed as nanomoles per gram wet weight [28].

### 2.6. Plasma Analysis

Fasting plasma insulin was determined using an ELISA kit (Crystal Chem). Plasma samples were analyzed for triglycerides, cholesterol, HDL-cholesterol, LDL-cholesterol, and NEFAs in a commercial laboratory (Comparative Clinical Pathology Services, Columbia, MO) on an automated clinical chemistry analyzer (AU680, Beckman-Coulter, Brea, CA) using the manufacturer's defined assays. Plasma IL-6, TNF*α* and MCP-1 were determined using a Milliplex mag mouse metabolic magnetic bead panel kit (Cat # MMHMAG-44K Millipore; Billerica, MA) that was analyzed using a Luminex MAGPIX system (Luminex Corporation; Houston, TX) and Milliplex Analyst software (Millipore; St. Charles, MO).

### 2.7. Statistics

The main effects of diet (AIN or SO) and genotype (WT or OB mice) were compared using a two-way ANOVA with main effect significance set at *P* < 0.05. When a significant interaction occurred between diet and genotype Bonferroni multiple comparisons was used to determine differences within groups at significance of *P* < 0.05. Values are reported as means ± standard error. 

## 3. Results

### 3.1. Sterculic Oil Decreases Tissue Desaturase Indices

**Figure 1:**
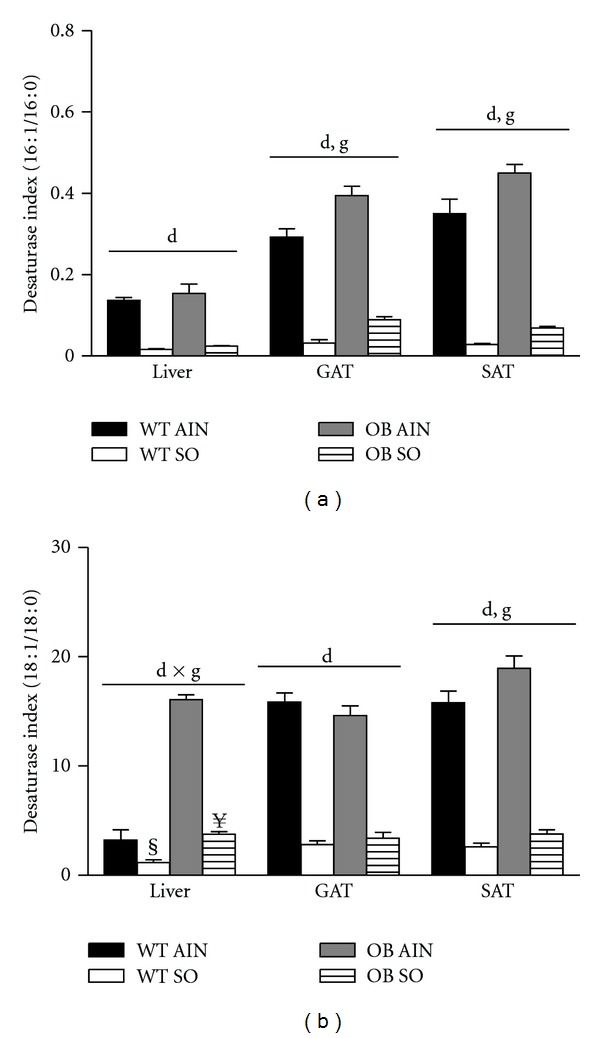
Sterculic oil reduces tissue desaturase indices. Desaturase indices (a) 16 : 1/16 : 0 and (b) 18 : 1/18 : 0 of liver, gonadal adipose (GAT), and subcutaneous adipose (SAT) tissues from wild-type (WT) and ob/ob (OB) mice receiving an AIN-93G diet (AIN) or a sterculic oil-supplemented diet (SO). Data are presented as means ± SE; *n* = 4–6 per group. Data were analyzed by two-way ANOVA and significance for the effects of diet (d), genotype (g), or their interaction (d × g) set at *P* < 0.05. When a significant interaction effect was found a Bonferroni multiple comparisons test was used to determine differences within groups. ^§^ *P* < 0.05 WT AIN versus WT SO; ^**¥**^ *P* < 0.05 OB AIN versus OB SO.

The desaturase indices are calculated as the product to substrate ratio of the fatty acids metabolized by the enzyme SCD1 (16 : 1/16 : 0 and 18 : 1/18 : 0) and are used as a proxy for the enzyme's activity [15, 29]. We observed an increase in at least one of the desaturase indices in the liver and adipose tissue of obese OB mice as compared to lean WT mice (Figure 1). These data are consistent with previous reports correlating increased SCD1 activity with an increase in obesity [15, 16, 29]. Regardless of phenotype, sterculic oil supplementation resulted in a dramatic reduction in the desaturase indices of liver and adipose tissue (Figure 1). Supplementation of sterculic oil increased the saturated fatty acid content of tissues as well as the content of sterculic and malvalic acids which are the cyclopropenoic fatty acids present in sterculic oil (Supplemental Tables 3, 4, and  5; available online at doi:10.5402/2012/947323).

### 3.2. General Animal Characteristics

**Figure 2:**
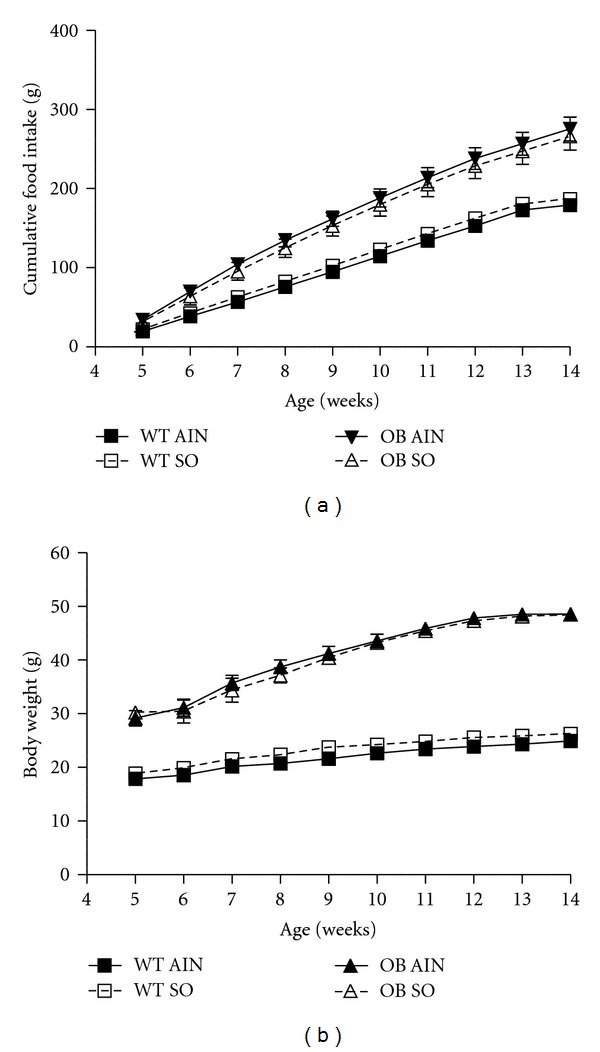
Sterculic oil supplementation did not alter food intake or body weight gain in ob/ob or wild-type mice. (a) Food intake and (b) body weight gain of wild-type (WT) and ob/ob (OB) mice receiving an AIN-93G diet (AIN) or a sterculic oil-supplemented diet (SO) over a 9 week experimental period. *n* = 6-7 per group; values are reported as mean ± SE.

Consistent with previous reports, ob/ob mice had increased food intake as well as body weight gain throughout the experiment compared to their wild-type counterparts (Figure 2). Sterculic oil supplementation did not affect food intake or body weight gain (Figure 2). Not surprisingly, the increased body weight of the ob/ob mice was accompanied by an increase in adipose tissue mass as compared to wild-type counterparts (Table 1). Interestingly, inhibition of SCD1 activity by sterculic oil supplementation did not result in a reduction in adipose tissue mass; rather a moderate increase was observed in the gonadal adipose tissue of OB SO mice as compared to OB AIN mice (Table 1). We observed no effect of sterculic oil on the mass of other adipose tissue depots in ob/ob or wild type mice (Table 1). However, liver mass was reduced (~23%) in ob/ob mice supplemented with sterculic oil (Table 1).

### 3.3. Sterculic Oil Improves Glucose Homeostasis in Obese Mice

**Figure 3:**
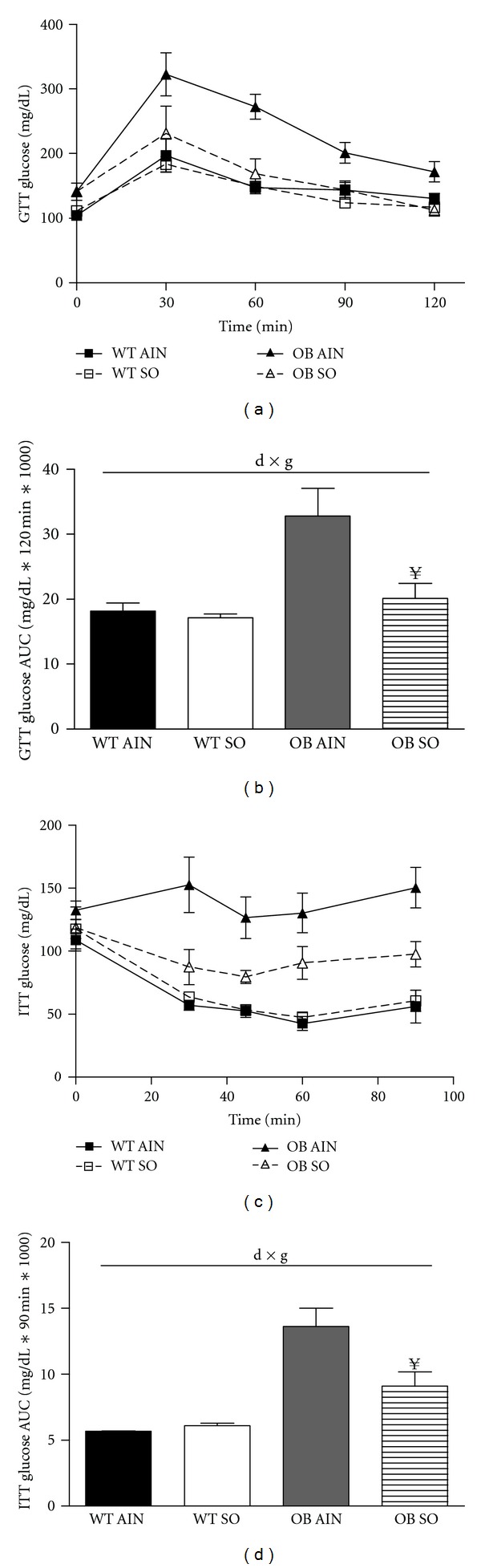
Sterculic oil supplementation improved glucose uptake and insulin sensitivity in obese ob/ob mice. (a) A glucose tolerance test (GTT) was performed in wild-type (WT) and ob/ob (OB) mice receiving an AIN-93G diet (AIN) or a sterculic oil-supplemented diet (SO) and (b) a corresponding blood glucose area under the curve (AUC) was calculated. (c) An interperitoneal insulin tolerance test (ITT) was performed in the same group of animals and blood glucose change over time was plotted and (d) blood glucose AUC calculations for the ITT. Treatment with SO improved glucose uptake and insulin sensitivity in ob/ob mice. Data are reported as mean ± SE; *n* = 4–7 per group. Data were analyzed by two-way ANOVA and significance for the effects of diet (d), genotype (g), or their interaction (d × g) set at *P* < 0.05. When a significant interaction effect was found a Bonferroni multiple comparisons test was used to determine differences within groups. ^**¥**^ *P* < 0.05 OB AIN versus OB SO.

In agreement with previous reports of impaired glucose metabolism, in response to a glucose tolerance test OB AIN mice had a significantly decreased ability to clear glucose (Figures 3(a) and 3(b)). Importantly, sterculic oil supplementation to obese ob/ob mice abrogated the increased blood glucose area under the curve despite a lack of difference in body weight (Figures 3(a) and 3(b)). However, glucose clearance was not enhanced in lean glucose tolerant mice (Figures 3(a) and 3(b)). In addition to improved glucose tolerance, we observed enhanced glucose lowering in response to insulin injection in OB SO mice as compared to OB AIN mice (Figures 3(c) and 3(d)). However, these improvements in glucose and insulin tolerance of OB SO mice occurred independent of changes in fasting glucose and insulin concentrations (Table 1). These improvements in glucose uptake and insulin sensitivity are consistent with previous reports of SCD1 inhibition [18, 30–32]; however our data differ from these studies as SO supplementation appears to dramatically inhibit the activity of the SCD1 enzyme without reducing SCD1 gene expression. In addition, the novelty of our findings is emphasized by a lack of change in adipose tissue mass which is a potential confounding variable in previous studies. 

### 3.4. Sterculic Oil Reduces Plasma Total Cholesterol Concentrations

Obesity and manipulation of SCD1 have both been reported to be associated with altered plasma lipid concentrations [33–35]. Likewise, we observed an increase in fasting concentrations of plasma triglycerides, total cholesterol, LDL-cholesterol, and HDL-cholesterol in obese ob/ob mice as compared to lean wild type mice (Table 2). Regardless of genotype, SO supplementation reduced total plasma cholesterol levels in both lean WT and obese ob/ob mice (Table 2). Fasting plasma NEFA concentrations were similar among all experimental groups (Table 2). Circulating concentrations of the cytokine IL-6 were also measured. While there was not a significant effect of diet on plasma IL-6 levels, we observed a large numeric increase in plasma IL-6 levels in the OB SO group (Table 2). Furthermore, circulating levels of TNF*α* and MCP-1 did not differ among groups (data not shown). 

### 3.5. Sterculic Oil Attenuates Hepatic Inflammatory and Lipogenic Gene Expression

**Figure 4:**
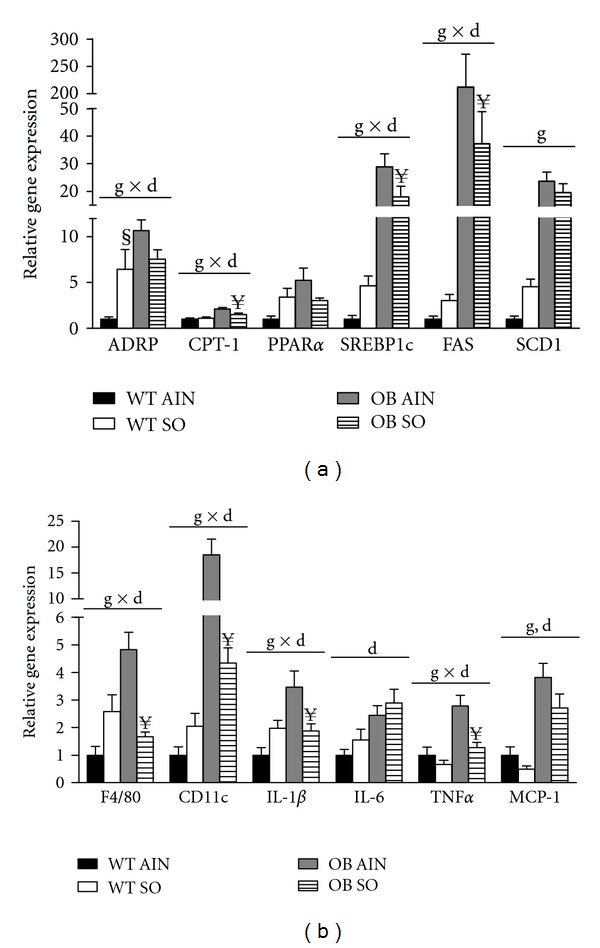
Sterculic oil alters hepatic inflammatory gene expression in ob/ob mice. Hepatic gene expression analysis was performed in wild-type (WT) and ob/ob (OB) mice receiving an AIN-93G diet (AIN) or a sterculic oil-supplemented diet (SO). (a) Hepatic lipogenic gene expression was unchanged by SO but elevated with obesity. (b) Obesity associated increases in hepatic inflammation were attenuated in part by SO supplementation. Data are presented as means ± SE; *n* = 6-7 per group. Data were analyzed by two-way ANOVA and significance for the effects of diet (d), genotype (g), or their interaction (d × g) set at *P* < 0.05. When a significant interaction effect was found a Bonferroni multiple comparisons test was used to determine differences within groups. ^§^ *P* < 0.05 WT AIN versus WT SO; ^**¥**^ *P* < 0.05 OB AIN versus OB SO.

Not unexpectedly, liver mass of obese ob/ob mice was significantly increased when compared to lean wild type mice (Table 1). This increase in mass was associated with an increase in hepatic triglyceride content. Interestingly, sterculic oil supplementation resulted in an ~23% reduction in liver mass of OB SO mice as compared to OB AIN mice (Table 1); however hepatic triglyceride content was not different between these two groups (Table 1). Sterculic oil did not influence liver mass or triglyceride content in lean wild type mice. Interestingly, despite a lack of difference in liver triglyceride content between OB AIN and OB SO mice, select lipogenic and oxidative genes were decreased by sterculic oil treatment (Figure 4(a)). 

 Although sterculic oil supplementation did not alter hepatic triglyceride content in the current study, we did observe changes in the profile of liver inflammatory genes. Consistent with trends in obesity, the livers of ob/ob mice had increased expression of markers for inflammatory cells and inflammatory cytokines as compared to lean WT mice. While hepatic triglyceride content was similar between OB AIN and OB SO mice, the profile of hepatic proinflammatory markers was attenuated with SO supplementation (Figure 4(b)). Overall, OB SO mice displayed a reversal in the inflammatory phenotype of the livers of OB AIN mice, which is associated with improved insulin sensitivity [36]. 

### 3.6. Sterculic Oil Alters Adipose Tissue Gene Expression

**Figure 5:**
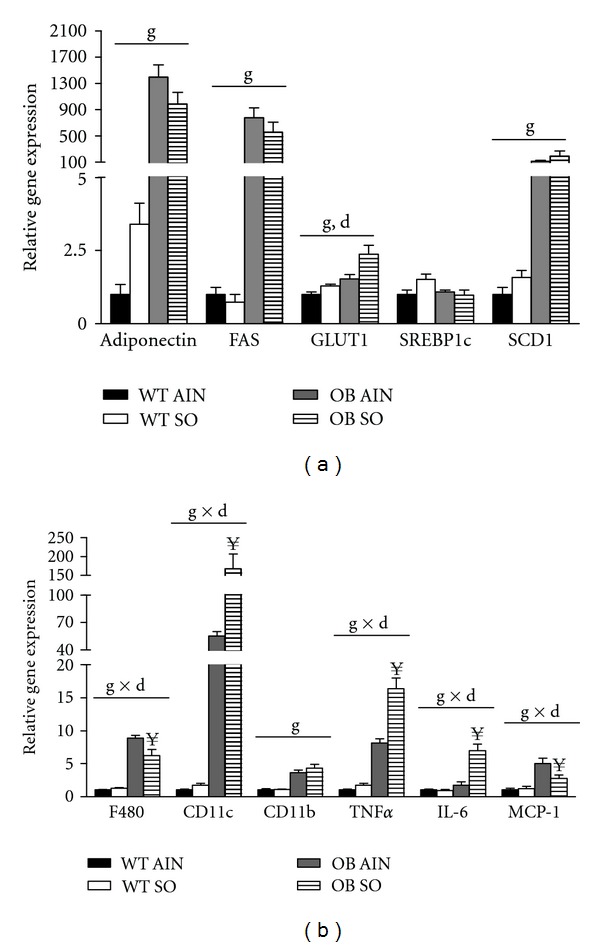
Sterculic oil supplementation increases expression of GLUT1 and markers of inflammation in gonadal adipose tissue of obese ob/ob mice. Gene expression analysis was performed in the gonadal adipose tissue of wild-type (WT) and ob/ob (OB) mice receiving an AIN-93G diet (AIN) or a sterculic oil-supplemented diet (SO). Relative mRNA expression of select (a) metabolic and (b) inflammatory genes. Data are presented as means ± SE; *n* = 6-7 per group. Data were analyzed by two-way ANOVA and significance for the effects of diet (d), genotype (g) or their interaction (d × g) set at *P* < 0.05. When a significant interaction effect was found a Bonferroni multiple comparisons test was used to determine differences within groups. ^**¥**^ *P* < 0.05 OB AIN versus OB SO.

**Figure 6:**
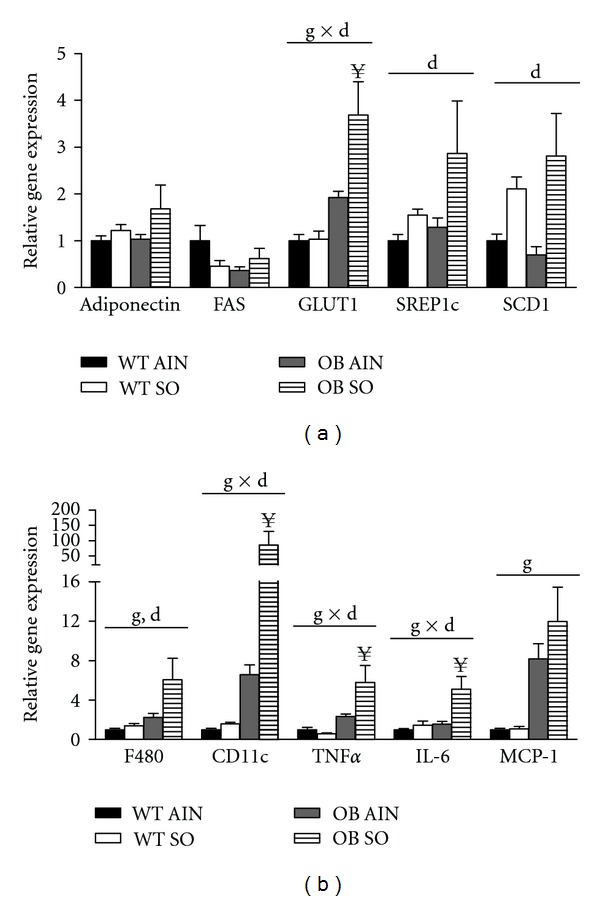
Sterculic oil associated changes in subcutaneous adipose tissue gene expression. Gene expression analysis was performed in subcutaneous adipose tissue isolated from wild-type (WT) and ob/ob (OB) mice receiving an AIN-93G diet (AIN) or a sterculic oil-supplemented diet (SO). Relative mRNA expression of select (a) metabolic and (b) inflammatory genes reveal a SO associated increase in GLUT1 expression in ob/ob mice and an obesity associated increase in select markers of inflammation. Data are presented as means ± SE; *n* = 6-7 per group. Data were analyzed by two-way ANOVA and significance for the effects of diet (d), genotype (g), or their interaction (d × g) set at *P* < 0.05. When a significant interaction effect was found a Bonferroni multiple comparisons test was used to determine differences within groups. ^**¥**^ *P* < 0.05 OB AIN versus OB SO.

As mentioned previously, the gonadal adipose tissue of OB SO mice was slightly larger than that of OB AIN mice while there was no difference in subcutaneous adipose tissue mass (Table 1). Histological analysis of adipose tissue sections revealed an effect of sterculic oil on adipocyte size in gonadal adipose tissue but not subcutaneous adipose tissue (Supplemental Figure  1; available online at doi:10.5402/2012/947323). This effect of sterculic oil on adipocyte hypertrophy may explain in part the increased gonadal adipose tissue mass observed in OB SO mice (Table 1). Complete and partial ablation of SCD1 have been reported to reduce adiposity as well as lipogenic gene expression in adipose tissue [31, 33, 37]. While the desaturase indices indicate a robust inhibition of SCD1 activity, gonadal adipose tissue gene expression of SCD1 and other lipogenic genes were not altered by sterculic oil supplementation in either wild type or ob/ob mice (Figure 5(a)). Conversely, SO supplementation resulted in an increase in expression of the lipogenic genes SREBP1c and SCD1 in subcutaneous adipose tissue regardless of geneotype (Figure 6(a)). Interestingly, sterculic oil supplementation resulted in a modest increase in GLUT1 gene expression in the gondal adipose tissue of WT and ob/ob mice as well as in the subcutaneous adipose tissue of ob/ob mice (Figures 5(a) and 6(a)). In previous studies, adipose tissue specific inhibition and/or ablation of SCD1 was reported to induce elevated expression of GLUT1 [38]. In addition, gene expression of markers of infiltrating/activated immune cells and inflammation was generally increased in the expanding gonadal and subcutaneous adipose tissue of the obese ob/ob mice (Figures 5(b) and 6(b)). Overall sterculic oil consumption was associated with an exacerbation of this response in obese ob/ob mice (Figures 5(b) and 6(b)).

## 4. Discussion

Many studies have displayed a critical role of SCD1's involvement in fatty acid metabolism and storage which is correlated with the development of obesity-induced insulin resistance [31, 37, 39, 40]. Therefore, SCD1 inhibitors have become one of the top ten mechanistic targets for potential pharmaceutical therapies for type 2 diabetes [13]. In an obese, metabolically challenged phenotype, reductions in SCD1 through genetic or pharmaceutical inhibition elicit improvements in insulin resistance, glucose clearance, and hypercholesterolemia as well as a reduction in adiposity [18, 31, 37, 40]. 

In the current study, we examined the metabolic changes associated with consumption of sterculic oil, a natural SCD1 inhibitor, in lean and obese mice. Our data demonstrate a dramatic reduction in the desaturase indices with sterculic oil supplementation, indicating a robust inhibition of SCD1 activity. However, unlike previous studies using genetic or pharmaceutical manipulation of SCD1 [32, 37, 38, 41], we did not observe reductions in SCD1 gene expression in the tissues investigated. The ability of sterculic oil to alter SCD1 activity but not expression may help to explain some of the discrepancies in metabolic outcomes between those previous studies and the current study. 

One of the key metabolic changes in our study was the improvement in glucose tolerance and insulin sensitivity that occurred with sterculic oil supplementation to obese ob/ob mice. Blood glucose area under the curve response for OB SO mice during the GTT and ITT was significantly improved, which is consistent with previous studies using SCD1 inhibition or ablation [31, 42, 43]. These previous studies have also reported reduced hepatic lipid accumulation, which is a predictor of insulin resistance [31, 32, 37]. Interestingly, in the present study, the improvement in glucose metabolism occurred independent of changes in hepatic triglyceride content although expression of hepatic lipogenic genes was reduced. Thus, our results suggest that a reduction in hepatic content of triglyceride is not a prerequisite for an improvement in glucose metabolism. In agreement with this, recent studies have also reported a disconnect between hepatic triglyceride content and hepatic insulin sensitivity and have suggested that intermediates of lipid metabolism such as diacylglycerides or ceramides are responsible for impaired insulin signaling [44–46]. Although not assessed in the current study, it is possible that sterculic oil may have altered liver lipid metabolism resulting in a reduction in these intermediates and improved glucose and insulin tolerance. Furthermore, hepatic triglyceride content is typically linked to an inflammatory response that is often closely correlated with obesity-associated insulin resistance [47–49]. While liver triglyceride content was similar between OB AIN and OB SO mice, hepatic markers of inflammation were decreased in OB SO mice as compared to OB AIN mice. This apparent disconnect between hepatic lipid content and inflammatory gene expression in the livers of OB SO mice may also help to explain the observed improvement in glucose tolerance in these animals. 

Increased adiposity is another predictor of insulin resistance. Previous studies have demonstrated that improvements in insulin signaling in mice lacking SCD1 coincide with a reduction in adiposity [18, 31]. Remarkably, sterculic oil improved glucose tolerance in obese ob/ob mice independent of a reduction in adiposity. Suppression of SCD1 activity also resulted in an altered fatty acid profile of adipose tissue that was characterized by an increased abundance of saturated fatty acids. *In vivo* and *in vitro* studies have shown that an increase in the availability of saturated free fatty acids mediates an inflammatory response resulting in the increased expression of inflammatory cytokines [50, 51]. The elevated concentration of saturated fatty acids within the adipose tissue may help explain the increases in gene expression of markers for activated immune cells and proinflammatory cytokines in OB SO mice as compared to the OB AIN group [50]. It is also possible that the adipocyte hypertrophy observed in the OB SO gonadal adipose tissue may also contribute to this effect.

We followed up on this observation and measured the plasma concentrations of circulating cytokines and chemokines. Despite the range of changes observed in adipose tissue gene expression, there were no significant changes in any of the measured cytokines (TNF-alpha, MCP-1, and IL-6). However, there was a substantial numeric increase in the plasma concentrations of IL-6 in OB SO mice. This increase was associated with significant increases in IL-6 gene expression in adipose tissue and liver of these animals. While IL-6 is traditionally considered a pro-inflammatory cytokine involved in the reduction of insulin sensitivity [52–54], there is separate evidence that disputes this dogma and suggests IL-6 may improve insulin sensitivity/glucose uptake [55–57]. Specifically, infusion of IL-6 during a hyperinsulinemic-euglycemic clamp has been reported to improved glucose uptake in humans [55], while *in vitro* studies have demonstrated that the addition of IL-6 in the presence of insulin enhances the translocation of GLUT4 to the cell surface, thus facilitating glucose uptake [55]. It is important to note that the protection from insulin resistance reported for SCD1 transgenic mice is associated with increased GLUT4 translocation; however, neither plasma concentrations nor tissue expression of IL-6 were reported in these studies [38, 58]. Therefore, our observation of increased IL-6 expression may provide an additional potential explanation for the observed improvement in glucose uptake and insulin sensitivity in the OB SO mice. 

While insulin signaling studies and quantification of GLUT4 translocation were beyond the scope of our study, we did observe an increase in GLUT1 gene expression with SO supplementation. This observation is consistent with results from Hyun et al. [38] demonstrating that white adipose tissue specific deletion of SCD1 in mice and pharmacological inhibition of SCD1 in 3T3-L1 adipocytes increased GLUT1 expression and glucose uptake. When taken in totality, inhibition of SCD1 has been reported to have varied effects on glucose uptake and/or glucose transporter expression suggesting that both tissue specific effects and the extent of SCD1 inhibition may influence regional glucose transporter expression and glucose metabolism [33, 38, 41]. A limitation of the current study is that muscle, a major sink for glucose uptake, was not collected. Therefore, we recognize that future studies utilizing hyperinsulinemic-euglycemic clamps and insulin signaling experiments will be required to definitively identify the target tissue(s) and mechanism(s) by which sterculic oil is improving glucose clearance. 

Overall, our data are consistent with previous reports demonstrating a robust inhibition of SCD1 activity with sterculic oil [9, 10, 59]. However, despite this reduction, the observed metabolic effects did not overlap completely with previous studies that have used alternative methods to ablate or attenuate SCD1. Nonetheless, we are the first to demonstrate that sterculic oil supplementation improves glucose and insulin tolerance in obese ob/ob mice. The exact mechanism of this improvement will require additional studies, but our data suggest this improvement may be due to increased GLUT1 expression in adipose tissue, increased expression of IL-6, or a decrease in hepatic inflammation. While SCD1 inhibition and sterculic oil consumption have been reported to have potentially adverse side effects associated with them [21–24], these were not a primary focus of the current study and no noticeable side effects were observed throughout the study. However, this will need to be explored in more detail in the future. It is noteworthy that the effects of sterculic oil consumption in lean mice appeared to be benign as glucose metabolism or any of the other afore mentioned parameters were not altered. Based on conversion factors described by Reagan-Shaw et al. [60], the daily human equivalent dose of sterculic oil consumed in this study was 34.3 mg/kg or 4.1 g/day for a 120 kg person suggesting that translation into human studies would be feasible. However, we acknowledge that additional studies will be required to verify and extend our findings before human studies can be fully considered. 

## Supplementary Material

Supplemental materials provided include the composition and fatty acid profile of experimental diets in addition to the fatty acid profile of liver, subcutaneous adipose tissue and gonadal adipose tissue from each experimental group. Histological images of representative subcutaneous and gonadal adipose tissue are included for all 4 treatment groups along with graphical presentation of adipocyte sizing data.

## Figures and Tables

**Table 1:** Characteristics of wild-type (WT) and ob/ob (OB) mice receiving an AIN-93G diet (AIN) or a sterculic oil-supplemented diet (SO)^1^.

					*P* value^2^		
	WT AIN	WT SO	OB AIN	OB SO	d	g	d × g
Body weight (g)	24.0 ± 0.5	25.2 ± 1.0	48.6 ± 1.1	48.5 ± 0.9	0.554	<0.001	0.471
Food intake^3^ (g/day)	2.7 ± 0.1	2.9 ± 0.1	4.1 ± 0.2	4.1 ± 0.4	0.582	<0.001	0.908
Tissue weights (g)							
Gonadal AT^4^	0.4 ± 0.0	0.5 ± 0.1	3.1 ± 0.1	3.8 ± 0.1^¥^	<0.001	<0.001	0.005
Subcutaneous AT	0.3 ± 0.0	0.4 ± 0.0	3.4 ± 0.1	3.5 ± 0.1	0.320	<0.001	0.828
Perirenal AT	0.1 ± 0.0	0.1 ± 0.0	2.1 ± 0.1	2.3 ± 0.1	0.375	<0.001	0.541
Liver	0.8 ± 0.0	1.0 ± 0.0	3.4 ± 0.1	2.8 ± 0.1^¥^	0.031	<0.001	<0.001
Liver TG^5^ (nmol/g wet wt)	12.0 ± 3.7	9.26 ± 2.8	25.2 ± 7.3	30.2 ± 8.3	0.626	<0.001	0.115
Blood glucose (mg/dL)	75.3 ± 5.7	82.4 ± 6.8	91.7 ± 6.6	94.8 ± 11.2	0.202	<0.05	0.552
Plasma insulin (ng/mL)	0.4 ± 0.0	0.3 ± 0.0	4.2 ± 0.1	6.7 ± 1.6	0.454	<0.001	0.471

^1^Data are presented as means ± SEM with *n* = 6-7 per group.

^2^Data were analyzed by 2-way ANOVA with diet (SO or AIN) as one factor and the phenotype (WT or OB) as the second factor. *P*-values are for an effect of diet (d), and effect of the genotype (g), or an interaction between the two (d × g). When a significant interaction between genotype and diet was found individual means were compared within groups by Bonferroni multiple comparisons.

^¥^ *P* < 0.05 OB AIN versus OB SO.

^3^Average daily food intake across the 9 week study.

^4^AT: adipose tissue.

^5^TG: triglyceride.

**Table 2:** Plasma characteristics of wild-type (WT) and ob/ob (OB) mice receiving an AIN-93G diet (AIN) or a sterculic oil-supplemented diet (SO)^1^.

					*P* value^2^		
	WT AIN	WT SO	OB AIN	OB SO	d	g	d × g
Plasma lipids (mg/dL)							
Triglyceride	117.1 ±12.0	87.5 ± 7.1	118.3 ± 3.1	122.2 ± 11.3	0.186	0.071	0.097
Total cholesterol	145.2 ± 6.6	136.5 ± 4.5	324.3 ± 7.6	290.0 ± 12.5	<0.05	<0.001	0.138
HDL-C	63.7 ± 8.1	68.7 ± 2.7	103.8 ± 4.2	92.0 ± 3.8	0.598	<0.001	0.165
LDL-C	21.2 ± 2.0	25.5 ± 3.2	72.8 ± 2.8	74.8 ± 3.5	0.301	<0.001	0.700
NEFA (mmol/L)	1.4 ± 0.1	1.2 ± 0.1	1.1 ± 0.1	1.2 ± 0.2	0.718	0.289	0.280
Plasma IL-6 (pg/mL)	96.9 ± 52.0	51.0 ± 15.8	67.8 ± 38.9	285.3 ± 52.0	0.454	0.047	0.138

^1^Data are presented as means ± SEM with *n* = 5–7 per group.

^2^Data were analyzed by 2-way ANOVA with diet (SO or AIN) as one factor and the phenotype (WT or OB) as the second factor. *P*-values are for an effect of diet (d), an effect of the genotype (g), or an interaction between the two (d × g).
