# Supplementary material for: Sterculic Oil, a Natural SCD1 Inhibitor, Improves Glucose Tolerance in Obese ob/ob Mice
Source: ISRN Endocrinol. 2012 Nov 14;2012:947323. doi: 10.5402/2012/947323 (PMC3504409; doi:10.5402/2012/947323)
Supplement: Supplementary file 1 — Supplemental materials provided include the composition and fatty acid profile of experimental diets in addition to the fatty acid profile of liver, subcutaneous adipose tissue and gonadal adipose tissue from each experimental group. Histological images of representative subcutaneous and gonadal adipose tissue are included for all 4 treatment groups along with graphical presentation of adipocyte sizing data. [file 947323.f1.docx]

Supplemental materials provided include the composition and fatty acid profile of experimental diets in addition to the fatty acid profile of liver, subcutaneous adipose tissue and gonadal adipose tissue from each experimental group. Histological images of representative subcutaneous and gonadal adipose tissue are included for all 4 treatment groups along with graphical presentation of adipocyte sizing data.

**Supplemental Table 1.** Diet composition of control and sterculic oil supplemented diets.

| Ingredient, % | Control^1^ | SO^2^ |
| --- | --- | --- |
| Cornstarch | 39.7 | 39.7 |
| Casein | 20.0 | 20.0 |
| Maltodextrin | 13.2 | 13.2 |
| Sucrose | 10.0 | 10.0 |
| Fiber | 5.0 | 5.0 |
| AIN-93G mineral mix | 3.5 | 3.5 |
| AIN-93G vitamin mix | 1.0 | 1.0 |
| L-cystine | 0.3 | 0.3 |
| Choline bitartrate | 0.3 | 0.3 |
| Soybean oil w/0.02% TB | 7.0 | 6.5 |
| Sterculic oil | 0.0 | 0.5 |
| ^1^ AIN-93G purified rodent diet  ^2^ AIN-93G diet containing sterculic oil (SO) at 0.5 g/100 g | | |

**Supplemental Table 2.** Fatty acid composition of control and sterculic oil (SO) diets.

| Fatty acid, % | Control^1^ | SO^2^ |
| --- | --- | --- |
| 16:0 | 10.59 | 10.83 |
| 18:0 | 3.94 | 3.99 |
| 18:1 (n-9) | 20.97 | 20.18 |
| 18:1 (n-7) | 1.41 | 1.36 |
| 18:2 (n-6) | 54.33 | 49.95 |
| 18:3 (n-3) | 6.92 | 6.12 |
| 18:CE^3^ | ND^4^ | 0.64 |
| 19:CE^5^ | ND | 3.81 |
| Other | 1.84 | 3.14 |
| ^1^ AIN-93G purified rodent diet  ^2^ AIN-93G diet containing sterculic oil at 0.5g/100g  ^3^ Malvalic acid (2-octyl-1-cyclopropene-1-octanoic acid)  ^4^ ND; not detected  ^5^ Sterculic acid (2-octyl-1-cyclopropene-1-heptanoic acid) | | |

| **Supplemental Table 3.**  Liver fatty acid profile of wild type (WT) and ob/ob (OB) mice receiving an AIN-93G diet (AIN) or a sterculic oil supplemented diet (SO).^1^ | | | | | | | |
| --- | --- | --- | --- | --- | --- | --- | --- |
|  |  |  |  |  | P-value^2^ | | |
|  | WT AIN | WT SO | OB AIN | OB SO | d | g | dxg |
| 14:0 | 0.61 ± 0.07 | 0.55 ± 0.03^§^ | 0.48 ± 0.01 | 0.67 ± 0.02 | 0.145 | 1.000 | <0.05 |
| 16:0 | 22.36 ± 0.59 | 30.06 ± 1.87 | 21.62 ± 0.47 | 31.24 ± 0.50 | <0.001 | 0.836 | 0.372 |
| 16:1 | 3.08 ± 0.19 | 0.46 ± 0.05 | 3.86 ± 0.40 | 0.77 ± 0.05 | <0.001 | 0.052 | 0.331 |
| 18:0 | 7.72 ± 2.33 | 11.79 ± 1.55 | 2.71 ± 0.12 | 9.47 ± 0.32 | <0.01 | <0.05 | 0.229 |
| 18:1 (n-9) | 19.11 ± 0.69 | 12.49 ± 1.07 | 43.48 ± 0.89 | 35.26 ± 1.14 | <0.001 | <0.001 | 0.276 |
| 18:1 (n-7) | 2.28 ± 0.65 | 0.70 ± 0.03^§^ | 5.74 ± 0.33 | 1.62 ± 0.10^¥^ | <0.001 | <0.001 | <0.01 |
| 18:2 (n-6) | 23.12 ± 3.83 | 24.86 ± 2.46 | 9.96 ± 0.74 | 10.84 ± 0.91 | 0.586 | <0.001 | 0.858 |
| 18:3 (n-3) | 1.44 ± 0.26 | 1.36 ± 0.22 | 1.22 ± 0.06 | 1.15 ± 0.07 | 0.688 | 0.241 | 0.956 |
| 18:CE^3^ | ND^4^ | 0.02 ± 0.01 | ND | 0.10 ± 0.01 |  |  |  |
| 19:CE^5^ | ND | 0.43 ± 0.18 | ND | 0.46 ± 0.03 |  |  |  |
| 20:4 (n-6) | 9.04 ± 1.99 | 6.47 ± 0.61 | 2.29 ± 0.13 | 1.65 ± 0.08 | 0.149 | <0.001 | 0.375 |
| 22:6 (n-3) | 5.06 ± 0.43 | 4.14 ± 0.37 | 1.85 ± 0.09 | 1.50 ± 0.12 | 0.053 | <0.001 | 0.354 |
| Other | 6.19 ± 0.73 | 6.68 ± 0.49 | 6.79 ± 0.28 | 5.28 ± 0.39 | 0.325 | 0.441 | 0.069 |
| Desaturation index |  |  |  |  |  |  |  |
| 16:1/16:0 | 0.15 ± 0.02 | 0.02 ± 0.00 | 0.14 ± 0.01 | 0.02 ± 0.00 | <0.001 | 0.3153 | 0.755 |
| 18:1/18:0 | 3.22 ± 0.93 | 1.17 ± 0.28^§^ | 16.09 ± 0.44 | 3.75 ± 0.24^¥^ | <0.001 | <0.001 | <0.001 |
| ^1^Data are presented as means ± SEM with n = 4-6 per group; means with different superscripts differ *P* < 0.05.  ^2^Data were analyzed by 2-way ANOVA with diet (SO or AIN) as one factor and the phenotype (WT or OB) as the second factor. P-values are for an effect of diet (d), and effect of the genotype (g), or an interaction between the two (dxg). When a significant interaction between genotype and diet was found individual means were compared within groups by Bonferroni multiple comparisons. ^§^P < 0.005 WT AIN vs. WT SO; ^¥^*P* < 0.05 OB AIN vs. OB SO.  ^3^Malvalic acid (2-octyl-1-cyclopropene-1-octanoic acid).  ^4^ND = not detectable.  ^5^Sterculic acid (2-octyl-1-cyclopropene-1-heptanoic acid). | | | | | | | |

| **Supplemental Table 4.**  Gonadal Adipose Tissue Fatty acid profile of wild type (WT) and ob/ob (OB) mice receiving an AIN-93G diet (AIN) or a sterculic oil supplemented diet (SO).^1^ | | | | | | | | | | | | | | | | | | | | | | |
| --- | --- | --- | --- | --- | --- | --- | --- | --- | --- | --- | --- | --- | --- | --- | --- | --- | --- | --- | --- | --- | --- | --- |
|  |  | |  | |  | |  | | | | | P-value^2^ | | | | | | | | | | |
|  | WT AIN | | WT SO | | OB AIN | | OB SO | | | | | d | | g | | | dxg | | | | | |
| 14:0 | 0.91 ± 0.03 | | 0.96 ± 0.05 | | 1.09 ± 0.05 | | 1.11 ± 0.08 | | | | 0.512 | | | <0.01 | | | 0.868 | | | | | |
| 16:0 | 16.87 ± 0.46 | | 24.08 ± 0.52 | | 18.84 ± 0.44 | | 23.32 ± 0.38 | | | | <0.001 | | | 0.217 | | | <0.05 | | | | | |
| 16:1 | 5.13 ± 0.22 | | 0.76 ± 0.21 | | 7.45 ± 0.54 | | 2.08 ± 0.19 | | | | <0.001 | | | <0.001 | | | 0.227 | | | | | |
| 18:0 | 1.93 ± 0.11 | | 8.15 ± 0.81 | | 2.29 ± 0.14 | | 8.81 ± 1.90 | | | | <0.001 | | | 0.266 | | | 0.866 | | | | | |
| 18:1 (n-9) | 30.97 ± 0.31 | | 22.01 ± 0.34^§^ | | 32.91 ± 0.77 | | 27.27 ± 0.67^¥^ | | | | <0.001 | | | <0.001 | | | <0.05 | | | | | |
| 18:1 (n-7) | 2.56 ± 0.07 | | 1.26 ± 0.04 | | 2.54 ± 0..13 | | 1.59 ± 0.05 | | | | <0.001 | | | 0.115 | | | 0.088 | | | | | |
| 18:2 (n-6) | 34.49 ± 0.63 | | 32.70 ± 0.60 | | 28.58 ± 1.03 | | 28.48 ± 1.55 | | | | 0.369 | | | <0.001 | | | 0.422 | | | | | |
| 18:3 (n-3) | 2.36 ± 0.09 | | 2.04 ± 0.15 | | 2.40 ± 0.18 | | 2.24 ± 0.20 | | | | 0.192 | | | 0.522 | | | 0.558 | | | | | |
| 20:4 (n-6) | 0.40 ± 0.03^§^ | | 0.22 ± 0.02 | | 0.46 ± 0.04 | | 0.50 ± 0.02 | | | | 0.054 | | | <0.001 | | | <0.005 | | | | | |
| 22:6 (n-3) | 0.20 ± 0.02 | | 0.16 ± 0.04 | | 0.26 ± 0.03 | | 0.25 ± 0.03 | | | | 0.397 | | | <0.05 | | | 0.632 | | | | | |
| 18:CE^3^ | ^ND4^ | | 2.62 ± 0.19 | | ND | | 0.89 ± 0.13 | | | |  | | |  | | |  | | | | | |
| 19:CE^5^ | ND | | 0.38 ± 0.03 | | ND | | 0.12 ± 0.03 | | | |  | | |  | | |  | | | | | |
| other | 4.18 ± 0.20 | | 4.68 ± 1.07 | | 3.21 ± 0.16 | | 3.35 ± 0.31 | | | | 0.525 | | | <0.05 | | | 0.718 | | | | | |
| desaturation index |  |  |  | | | | |  |  | | | |  |  |  |  | |  |  |  |  |  |
| 16:1/16:0 | 0.29 ± 0.02 | | 0.03 ± 0.01 | 0.39 ± 0.02 | | 0.09 ± 0.01 | | | | <0.001 | | | | <0.001 | | | 0.274 | | | | | |
| 18:1/18:0 | 15.86 ± 0.86^a^ | | 2.80 ± 0.35 | 14.62 ± 0.90 | | 3.41 ± 0.49 | | | | <0.001 | | | | 0.695 | | | 0.259 | | | | | |
| ^1^Data are presented as means ± SEM with n = 4-6 per group; means with different superscripts differ *P* < 0.05.  ^2^Data were analyzed by 2-way ANOVA with diet (SO or AIN) as one factor and the phenotype (WT or OB) as the second factor. P-values are for an effect of diet (d), and effect of the genotype (g), or an interaction between the two (dxg). When a significant interaction between genotype and diet was found individual means were compared within groups by Bonferroni multiple comparisons. ^§^P < 0.005 WT AIN vs. WT SO; ^¥^*P* < 0.05 OB AIN vs. OB SO.  ^3^ Malvalic acid (2-octyl-1-cyclopropene-1-octanoic acid).  ^4^ ND = not detectable.  ^5^ Sterculic acid (2-octyl-1-cyclopropene-1-heptanoic acid). | | | | | | | | | | | | | | | | | | | | | | |

| **Supplemental Table 5.** Subcutaneous adipose tissue fatty acid profile of wild type (WT) and ob/ob (OB) mice receiving an AIN-93G diet (AIN) or a sterculic oil supplemented diet (SO).^1^ | | | | | | | |
| --- | --- | --- | --- | --- | --- | --- | --- |
|  |  |  |  |  | P-value^2^ | | |
|  | WT AIN | WT SO | OB AIN | OB SO | d | g | dxg |
| 14:0 | 1.00 ± 0.07 | 0.99 ± 0.03 | 1.16 ± 0.06 | 1.16 ± 0.06 | 0.857 | <0.05 | 0.985 |
| 16:0 | 15.83 ± 0.61 | 22.95 ± 0.17 | 16.08 ± 0.41 | 22.92 ± 0.63 | <0.001 | 0.786 | 0.745 |
| 16:1 | 5.53 ± 0.61 | 0.63 ± 0.07 | 7.27 ± 0.46 | 1.58 ± 0.07 | <0.001 | <0.01 | 0.384 |
| 18:0 | 2.06 ± 0.15 | 9.09 ± 1.28 | 1.81 ± 0.10 | 7.79 ± 1.13 | <0.001 | 0.281 | 0.458 |
| 18:1 (n-9) | 31.66 ± 0.19 | 22.17 ± 0.49^§^ | 33.76 ± 0.32 | 27.86 ± 0.25^¥^ | <0.001 | <0.001 | <0.001 |
| 18:1 (n-7) | 2.43 ± 0.09 | 1.30 ± 0.09 | 2.87 ± 0.06 | 1.57 ± 0.1 | <0.001 | <0.001 | 0.325 |
| 18:2 (n-6) | 34.48 ± 0.48 | 32.52 ± 0.82 | 30.45 ± 0.99 | 29.98 ± 1.65 | 0.256 | <0.01 | 0.478 |
| 18:3 (n-3) | 2.06 ± 0.04 | 1.94 ± 0.12 | 2.33 ± 0.05 | 2.07 ± 0.13 | <0.05 | <0.05 | 0.402 |
| 18:CE^3^ | ND^4^ | 2.92 ± 0.24 | ND | 1.25± 0.16 |  |  |  |
| 19:CE^5^ | ND | 0.41 ± 0.03 | ND | 0.18 ± 0.02 |  |  |  |
| 20:4 (n-6) | 0.32 ± 0.03 | 0.22 ± 0.01 | 0.40 ± 0.02 | 0.32 ± 0.01 | <0.01 | <0.01 | 0.592 |
| 22:6 (n-3) | 0.14 ± 0.01 | 0.19 ± 0.02 | 0.18 ± 0.02 | 0.17 ± 0.01 | 0.427 | 0.399 | 0.066 |
| other | 4.50 ± 0.19 | 4.70 ± 0.37 | 3.67 ± 0.09 | 3.16 ± 0.08 | 0.429 | <0.001 | 0.092 |
| Desaturation index |  |  |  |  |  |  |  |
| 16:1/16:0 | 0.35 ± 0.049 | 0.03 ± 0.00 | 0.45 ± 0.02 | 0.07 ± 0.00 | <0.001 | <0.05 | 0.267 |
| 18:1/18:0 | 15.80 ± 1.06 | 2.60 ± 0.37 | 18.93 ± 1.16 | 3.76 ± 0.42 | <0.001 | <0.05 | 0.342 |
| ^1^Data are presented as means ± SEM with n = 4-6 per group; means with different superscripts differ *P* < 0.05.  ^2^Data were analyzed by 2-way ANOVA with diet (SO or AIN) as one factor and the phenotype (WT or OB) as the second factor. P-values are for an effect of diet (d), and effect of the genotype (g), or an interaction between the two (dxg). When a significant interaction between genotype and diet was found individual means were compared within groups by Bonferroni multiple comparisons. ^§^P < 0.005 WT AIN vs. WT SO; ^¥^*P* < 0.05 OB AIN vs. OB SO.  ^3^ Malvalic acid (2-octyl-1-cyclopropene-1-octanoic acid).  ^4^ ND = not detectable.  ^5^ Sterculic acid (2-octyl-1-cyclopropene-1-heptanoic acid). | | | | | | | |

Supplemental Figure 1. Sterculic oil supplementation is associated with alterations in adipocyte size in gonadal but not subcutaneous adipose tissue. Adipocyte cell size in the (A) gonadal adipose tissue is increased with obesity and sterculic oil treatment, whereas adipocyte size was only increased with obesity in (B) subcutaneous adipose tissue. These differences can be seen in histological sections of the (C) gonadal and (D) subcutaneous adipose tissue. Data are presented as means ± SE; n = 6-7 per group; Data were analyzed by two-way ANOVA and significance for the effects of diet (d), genotype (g) or their interaction (dxg) set a P < 0.05. When a significant interaction effect was found a Bonferroni multiple comparisons test was used to determine differences within groups. **^§^***P* < 0.05 WT AIN vs. WT SO; **^¥^** *P* < 0.05 OB AIN vs. OB SO.
